# Hints for the Sick Room

**Published:** 1889-03-23

**Authors:** M. M. Basil

**Affiliations:** Author of "The Commoner Accidents to Life and Limb"


### Hints for the Sick-room.

By M. M. Basil, M.A., M.B., C.M., Author of "The Commoner Accidents to Life and Limb."

*(Continued from p. 385.)*

### BEDSIDE MANIPULATIONS.

The effect of certain medicines on the urinary functions must be watched and reported early. Turpentine and cantharides, after absorption by the skin or other channels, may produce painful or frequent micturition, or traces of blood in the urine. These symptoms should be noted, the medicine discontinued, and the fact reported at the next visit. Turpentine imparts, in addition, a peculiar smell to the urine. Carbolic acid gives a smoky tint to the urine, and causes pain or uneasiness during micturition. When this agent is used freely in dressing wounds its action should be carefully observed. Rhubarb and santonine impart a rich orange-yellow colour to the secretion when its reaction is acid. The colour, however, becomes deep red if the urine is alkaline. It is important to bear in mind these colour reactions, as they alarm the patient and his friends very much, and the anxiety is increased if the nurse looks puzzled or nervous.

The detailed examination of the urine cannot be taken up here, but some cautions may be mentioned. When a quantity is to be poured into the urine glass for determining the specific gravity, room should be allowed for displacement upwards of the fluid by the urinometer. Annoyance is often caused by filling the glass nearly to the top. In boiling urine the mouth of the test-tube should always be directed away from self and neighbours. Few run the risk of scalding themselves a second time; but, unfortunately, the same immunity from second attacks is not enjoyed by people standing near the operator. Remember that the strong acids and alkalis used in testing urine are corrosive substances; do not grasp the stopper of such bottles. In warm weather point the mouth of the nitric acid bottle away from yourself before opening it. A jet of corrosive drops may be spurted out on the removal of the stopper if the bottle has been jerked about much or left airtight for some time.

The observation of the evacuations is of importance in all cases of serious disease. The nurse must be particularly well informed in this subject so as to notice all that is important to the study of the case. In a hospital it is important to get rid of the fœtid, often infectious, alvine excretions as soon as possible. The privy air which is generated at no great distance from the sick-room by saving the stools for medical inspection is a dangerous nuisance. The nurse should, therefore, qualify herself to observe the essential points, and get rid of the excretions at once. In cases where they must be saved for inspection it is advisable to pour some colourless disinfectant, such as strong carbolic acid lotion or sanitas, into the vessel. Some of the important points which must be observed are the amount, frequency, colour, consistence, smell, the presence of foreign bodies, such as worms, undigested food, gallstones, casts, polypi, and shreds of tissue. Blood may be found in the stools as red streaks, red fluid, in clots, or it may be much altered, and appear as a tarry fluid (melæna). The last condition should be reported at once, especially during typhoid fever or after severe burns. Dark discoloration of the motions may also be due to other causes: preparations of iron, bismuth, lead, antimony, and silver, darken the stools in various degrees. Hæmotoxylin, or logwood, reddens it, calomel in certain quantities gives it a grass-green appearance (calomel stools), well marked in children. It is important to remember that this chopped-spinach appearance of stool may be due to other causes, particularly in children. The pea-soupy stools of typhoid fever will be spoken of later on. In some cases the motions have a pipe-clay or ribbon shape. The evacuation of the bowels may be accompanied with pain, or pain may precede or follow the function.

The observation of vomited matter and the time of vomiting are also of importance. The most striking alteration here is the hare-soupy appearance of the vomit in ulcer of the stomach. All vomited matter of suspicious appearance should be saved for inspection, so should urinary calculi, gallstones, stones from the ears and nose, objects expelled from the vagina, trachea, bowels, and all objects removed by operation. You should bear in mind that specimens of the vomit may become of considerable medico-legal importance; indeed, any case under treatment may become a medico-legal one, and you may be called upon to give evidence. You should, therefore, keep a careful eye on facts coming under observation, without making yourselves officious.

The condition of the skin must not be overlooked. It may be dry, moist, or clammy; hot, pungent, or cold. It may be seriously discoloured or in a state of desquamation, peeling in minute branny scales (measles), or in large flakes (scarlatina). Eruptions must be observed minutely. It is important to note down the time of their appearance, the place where they are first seen, their general character, the changes they undergo, and whether they cause itchiness or not. The state of the skin peculiar to erysipelas and lymphatic inflammation must be observed early, so as to segregate the patient at once, and check the spread of an infectious disease. Abscesses and bruises on the body must be carefully looked for soon after a patient is admitted into hospital In absence of such precaution, those who are inclined to mischief may have it in their power to shoulder the blame of their infliction on the wrong individual. Similarly, all accidental injuries, falls out of bed or during fits, should be reported at once, because even apparently slight injuries may lead to serious results after several days. If the circumstances of the accident cannot be clearly investigated from lapse of time, unjust blame may be attached to persons who are really innocent.

Profuse perspiration of the whole body, or any part of it, such as the head, should be observed as to time and duration of occurrence. Rigors or severe shaking fits must have their time of onset, duration, and character noted down. The temperature of the patient should always be taken during a rigor. It will most frequently be found that while he shakes violently and complains of chilliness his temperature is really several degrees above the normal, so that he shivers in high fever. Severe pains should be observed as to their time of onset, duration, seat and direction in which they spread. The duration of sleep, whether it is sound, heavy, or disturbed, the presence of coma, unconsciousness, or mere stupor, should be noticed. The occurrence of delirium must be reported immediately.

The well-trained nose is of considerable importance in the sick room. It would be advisable not to be led by the nose alone in deciding the character of any disease; but the smell of smallpox, typhus fever, acute rheumatism, favus of the scalp, gangrene of the lungs, bronchi-ectases, and advanced cancer, are, unfortunately, but too conclusive to many nasal organs. The smell of opium and belladonna are also characteristic, and should be thoroughly familiar to you. Of course there are people who cannot discriminate smells, as there are people who are colour-blind; but this does not shake the confidence generally placed in our sense of smell or of sight.

The general posture assumed by a patient is worthy of observation, as it throws much light on the nature and seat of the disease, and more especially on the satisfactory nursing of the case. In many painful abdominal and pelvic diseases the knees are drawn up so as to relieve tension of the painful parts as much as possible. The nurse may, in such cases, materially help the patient, and add to her comfort by supporting the knees and shoulders with firm pillows. In painful diseases of the chest (pleurisy) the patient generally avoids the sore side at first, but as the disease leads on to collection of a large amount of liquid round the lung, the patient comes to lie neither quite on the diseased side nor flat on the back, but diagonally, that is, towards but not upon the affected side. It is important for you to remember this, for should such a patient become drowsy, comatose, or be brought under the influence of chloroform, turning her completely to the healthy side will add largely to the risks of death by asphyxia. The patient has practically one lung to depend upon for her breathing, and pressure upon that lung by a bag of fluid is equivalent to a rope round the neck.

Some patients can breathe with comfort only in a sitting or semi-sitting position. Lowering the shoulders embarrasses the respiration at once. In these cases means must be provided to enable the patient to sleep in a semi-sitting posture. Death may be accelerated by gradual lowering of the shoulders during sleep or in narcosis.

*(To be continued.)*

